# *Introspective Minds*: Using ALE Meta-Analyses to Study Commonalities in the Neural Correlates of Emotional Processing, Social & Unconstrained Cognition

**DOI:** 10.1371/journal.pone.0030920

**Published:** 2012-02-03

**Authors:** Leonhard Schilbach, Danilo Bzdok, Bert Timmermans, Peter T. Fox, Angela R. Laird, Kai Vogeley, Simon B. Eickhoff

**Affiliations:** 1 Max-Planck-Institute for Neurological Research, Cologne, Germany; 2 Department of Psychiatry, University of Cologne, Cologne, Germany; 3 Institute of Neuroscience and Medicine (INM-1), Research Center Juelich, Juelich, Germany; 4 Department of Psychiatry, University of Aachen, Aachen, Germany; 5 Research Imaging Center, UT Health Science Center, San Antonio, Texas, United States of America; 6 Institute of Neuroscience and Medicine (INM-3), Research Center Juelich, Juelich, Germany; 7 Cognitive Neuroscience Group, Institute of Clinical Neuroscience and Medical Psychology, University of Duesseldorf, Duesseldorf, Germany; Bellvitge Biomedical Research Institute-IDIBELL, Spain

## Abstract

Previous research suggests overlap between brain regions that show task-induced deactivations and those activated during the performance of social-cognitive tasks. Here, we present results of quantitative meta-analyses of neuroimaging studies, which confirm a statistical convergence in the neural correlates of social and resting state cognition. Based on the idea that both social and unconstrained cognition might be characterized by introspective processes, which are also thought to be highly relevant for emotional experiences, a third meta-analysis was performed investigating studies on emotional processing. By using conjunction analyses across all three sets of studies, we can demonstrate significant overlap of task-related signal change in dorso-medial prefrontal and medial parietal cortex, brain regions that have, indeed, recently been linked to introspective abilities. Our findings, therefore, provide evidence for the existence of a core neural network, which shows task-related signal change during socio-emotional tasks and during resting states.

## Introduction

Previous research suggests overlap between those brain regions that show task-induced deactivations across a wide range of different fMRI studies (the so-called ‘default mode network’ or ‘resting state’ of brain function; e.g. [1]) and brain regions, which are activated during the performance of social-cognitive tasks (often referred to as the ‘mentalizing network’, e.g. [2], [3]). The set of brain areas thus implicated includes medial prefrontal cortex, posterior cingulate cortex/precuneus and the temporo-parietal junction. The observed similarities have led to the suggestion that a relationship may exist between the physiological baseline of the human brain and a psychological baseline or predisposition for social cognition, which may be characterized by a reliance on internally-directed attention or introspective processes (cf. [4]–[6]). A close relationship is also thought to exist between activity change in the ‘default mode network’ and emotional processing (e.g. [7]–[9]), which could be related to a close link between emotional experiences and introspection (e.g. [10]). In this respect, it has been suggested that introspective competence may initially emerge from an infant's social interactions with others and the emotional experiences made in this context (e.g. [11]). Consequently, our notion of introspection is one, which sees introspective abilities as a capacity to attend to one's own feelings and/or thoughts, which is to an important degree forged in the process of social interaction. In the context of neuroimaging studies into socio-emotional and resting state cognition, we see introspection, thus defined, as a possible ‘common denominator’ of both socio-emotional and resting state cognition.

In order to address whether social-cognitive (*hereafter:* SOC), resting state (*hereafter:* DMN) and emotional processing (*hereafter:* EMO) rely on a common core network of brain regions we took the following approach: we used the activation likelihood estimation (ALE) approach, which allows to statistically investigate the convergence of published neuroimaging findings on activations or deactivations to delineate brain regions, that are consistently activated by social-cognitive and emotional tasks, as well as those that consistently deactivate following the engagement in a wide range of other experimental tasks [12]–[14].

In a first step, the neural correlates of these different processes (SOC, EMO and DMN) were analyzed separately. In a second step, conjunction analyses were performed to reveal brain areas, which show consistent task-related signal change across two sets of studies. Given the phenomenal and conceptual similarities of social-cognitive and emotional processes and their allegedly comparable importance for the understanding of other minds (e.g. [15]), we used the conjunction of SOC ∩ EMO to validate our approach and map out commonalities in task-related brain activity between the two independent sets of studies. In order to investigate neurofunctional overlap between social and unconstrained cognition, we performed the conjunction of SOC ∩ DMN. Lastly, and most important for the objective of our study, a triple conjunction analysis was carried out to investigate convergence across all three types of studies (SOC ∩ EMO ∩ DMN) based on the assumption that introspective processes could be equally relevant across all studies. In order to investigate whether the core neural network common to all three sets of studies shows overlap with findings from a recent study by Fleming et al., which investigated the relationship of gray matter volume differences and introspective abilities [16], we performed a minimum conjunction analysis as the intersection of the statistical maps. In the study by Fleming and colleagues participants were asked to perform a (two interval, forced choice) visual judgment task presented near (individually determined) sensory thresholds and to provide ratings of confidence in their decisions after each trial. Introspective ability was determined at an individual level through the construction of type II receiver operating characteristic (ROC) curves, which provide a measure of the ability to link confidence to perceptual performance, i.e. of being able to (subjectively) report how well one is (objectively) doing the task.

## Methods

### Data used for the meta-analysis

Functional imaging experiments for the meta-analyses were obtained from the BrainMap database [12], which contained, at the time of analysis, the location of reported activation foci and associated meta-data of approximately 10,000 neuroimaging experiments. Of these, only databased functional magnetic resonance (fMRI) and positron emission tomography (PET) studies that reported functional mapping experiments were considered. The *inclusion criteria* comprised considering only data from neurotypicals (i.e. healthy subjects), exclusive consideration of studies that report coordinates provided in a standard anatomical reference space (Talairach/Tournoux or MNI) and reliance on full-brain coverage (versus analyses based on regions of interest (ROIs) or functional localizers). The *exclusion criteria* consisted in non-consideration of neuroimaging experiments that investigate age, gender, patient groups or drug effects. For retrieval of the relevant experiments, the BrainMap database was filtered based on the metadata of the archived experiments that describe the experimental paradigm used in the respective experiment as well as the specific mental process isolated by a given statistical contrast. Behavioural domains (BD) include the main categories of cognition, action, perception, emotion, interoception, as well as their related subcategories. The respective paradigm classes (PC) classify the specific task employed (a complete list of BDs and PCs can be found at http://brainmap.org/scribe/). The BDs and PCs together constitute a complete cognitive taxonomy for the conceptual distinction of mental processes conceived by leaders of the neuroscientific community [17]. Furthermore, labels denoting the characterization of the reported coordinates as task-dependent activation or deactivation are provided by the BrainMap metadata for each experiment.

In the current analysis of the DMN, we included all experiments that reported deactivations relative to a low-level (resting) baseline, i.e. all experiments providing coordinates for regions that decreased their activity when subjects were engaged in an externally structured task (DMN: n = 533), while excluding those studies, which were part of the other individual meta-analyses. That is, the individual meta-analyses were based on independent pools of experiments. Furthermore, we profited from the BrainMap taxonomy, to identify the sub-pools of the databased neuroimaging experiments that have been labelled as being closely related to emotional and social processing, respectively. Here, the considered studies on emotional processing are defined as pertaining to “the mental faculty of experiencing an affective state of consciousness such as joy, sorrow, fear, hate”. In particular, facial emotion is thought to be an evolutionarily shaped adaption that allows for the efficient transmission of affective states [18]. Experimental psychology has later detailed that experiencing one's own and witnessing others' emotions is closely related to autonomic arousal [19], [20]. Importantly, a number of facial emotions are known to be interculturally stable [21], which underlines the psychological importance of automatically sharing arousing affective states, that is, emotions. The considered studies on social processing, on the other hand, are defined as pertaining to “the mental faculty associated with how people process social information, especially its encoding, storage, retrieval, and application to social situations”. Notably, even stimuli with simple geometrical shapes are perceived as human-like agents if they enact social situations (e.g. [22]). Also from a developmental perspective, it is known that neurotypically developing infants have a preference to attend to social cues (e.g. [23]). Likewise, it is known that memory encoding and retrieval is biased towards social content, such as interactions and relationships (e.g. [24], [25]), which underscores the psychological distinctness of social-cognitive processes. In particular when considering the focus of the current investigation, i.e., to assess the convergence between social cognition and unconstrained processing in the “default mode”, it is important to note, that the employed definition of social-cognitive processes did not include primarily self-referential processing such as self-evaluations/-descriptions. That is, our meta-analysis on social-cognitive processing only included studies dealing with the impression and evaluation of other persons, not the self. This restriction should be important, as it is well-known that processing of the self (or close others) shows is supported brain regions that are discernable from those sustaining social-cognitive processing focusing on other, unfamiliar people (cf. [26], [27]). Taken together, based on the BrainMap meta-data, we identified all experiments corresponding to contrasts isolating emotional processing (EMO: n = 1474) as well as those experiments that probed social-cognitive processes (SOC: n = 75). For those two analyses (EMO, SOC) only task-associated activations were considered.

### Meta-analysis algorithm

Meta-analyses were carried out using the revised version [28] of the activation likelihood estimation (ALE) approach for coordinate-based meta-analysis of neuroimaging results [29], [30]. The algorithm aims at identifying areas showing a statistical convergence of reported activations across different experiments. Inference is then based on the assessment if this clustering is higher than expected under the null distribution of a random spatial association. The key idea behind ALE is to treat the reported foci not as single points, but rather as centers for 3D Gaussian probability distributions capturing the spatial uncertainty associated with each focus. The width of these uncertainty functions was determined based on empirical data on the between-subject and between-template variance, which represent the main components of this uncertainty. Importantly, the applied algorithm weights the between-subject variance by the number of examined subjects per study, accommodating the notion that larger sample sizes should provide more reliable approximations of the ‘true’ activation effect and should therefore be modelled by ‘tighted’ Gaussian distributions [28].

The probabilities of all activation foci in a given experiment were combined for each voxel, resulting in a modelled activation map (MA map). Taking the union across these MA maps yields voxel-wise ALE scores describing the convergence of results at each particular location. Since neurophysiologically, neuronal effects should predominantly be localized within the grey matter, all analyses were restricted to those voxels where a probability of at least 10% for grey matter could be assumed based on the International Consortium of Brain Mapping (ICBM) tissue probability maps. To distinguish ‘true’ convergence between studies from random convergence, i.e., noise, ALE scores were compared to an empirical null-distribution reflecting a random spatial association between experiments. Hereby, a random-effects inference is invoked, focusing on inference on the above-chance convergence between studies, not clustering of foci within a particular study. Computationally, deriving this null-hypothesis involved sampling a voxel at random from each of the MA maps and taking the union of these values in the same manner as done for the (spatially contingent) voxels in the true analysis. The p-value of a ‘true’ ALE was then given by the proportion of equal or higher values obtained under the null-distribution. The resulting non-parametric p-values for each meta-analysis were thresholded at a cluster-level family wise error (FWE) corrected threshold of p<0.05 and transformed into Z-scores for display.

Conjunction analyses between the separate meta-analyses were carried out by calculating a voxel-wise minimum statistic [31]. Computationally, this is equivalent to determining the intersection between the thresholded meta-analyses results (cf. [32]). Results were thus significant in individual analyses at a corrected p<0.05. The resulting areas were anatomically labelled by reference to probabilistic cytoarchitectonic maps of the human brain [33], [34].

In order to formally test whether the core ‘introspection network’ - represented by the results of our triple conjunction analysis (SOC ∩ EMO ∩ DMN) – shows anatomical overlap with the findings from the study by Fleming and colleagues [16], which investigated the relationship of grey matter differences and introspective abilities, a minimum conjunction analysis was computed as the intersection of the thresholded statistical parametric maps.

## Results

### Individual meta-analyses

#### ‘Social-cognitive network’ (SOC)

**Figure 1:**
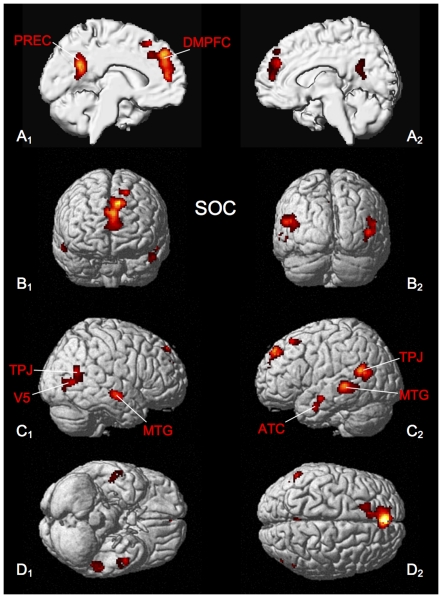
Significant results of the ALE meta-analysis for social cognition tasks (SOC). All results are displayed on the left and right lateral surface view, the anterior/posterior and dorsal/ventral view of the Montreal Neurological Institute (MNI) single subject template. ATC: anterior temporal cortex, DMPFC: dorso-medial prefrontal cortex, MTG: middle temporal gyrus, PCC: posterior cingulate cortex, PREC: precuneus, TPJ: temporo-parietal junction.

Consistent activation across the social-cognitive tasks was found in left dorso-medial prefrontal cortex (DMPFC), the left precuneus, bilateral middle temporal gyrus, anterior temporal cortex (ATC) and the temporo-parietal junction (TPJ) area as well as the left superior frontal gyrus (Figure 1, Table 1).

**Table 1:** Activation peaks of ALE meta-analysis of the neural correlates of social cognition tasks (SOC).

Macroanatomical location	MNI coordinates			Cluster size	Maximum z-score
	x	y	z		
Left Superior Medial Gyrus	4	46	32	1083	7.40
Left Precuneus	−6	−52	28	642	6.67
Left Middle Temporal Gyrus	−48	−59	18	456	5.22
Left Middle Temporal Gyrus	−53	−38	0	385	5.56
Right Middle Temporal Gyrus	48	−65	10	259	5.42
Right Middle Temporal Gyrus	52	−13	−10	236	3.98
Left Middle Temporal Gyrus	−50	−5	−19	179	4.60
Left Superior Frontal Gyrus	−17	25	55	116	4.31

All peaks were assigned to the most probable brain area by using the SPM Anatomy Toolbox.

#### ‘Emotional processing network’ (EMO)

**Figure 2:**
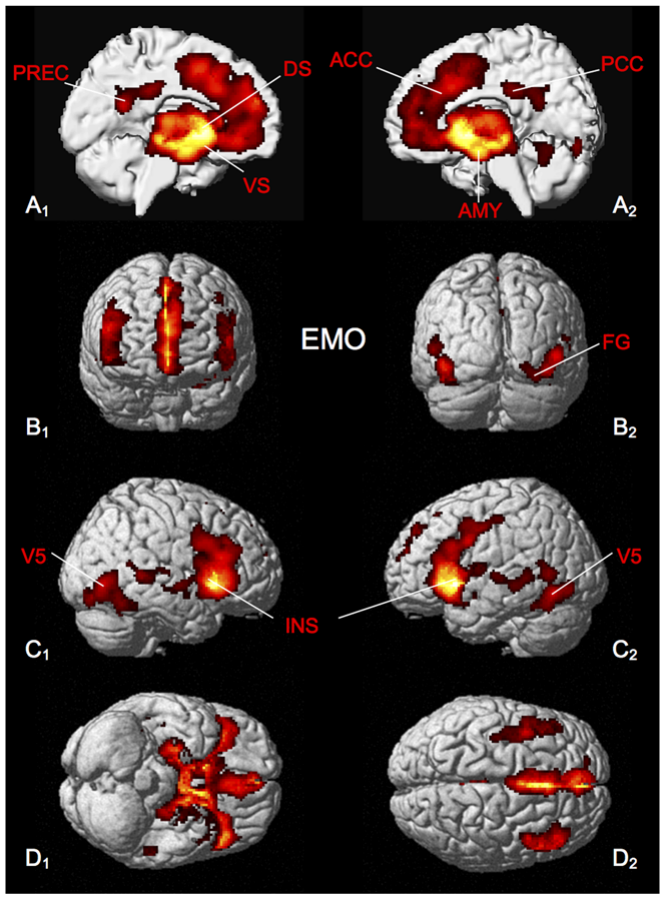
Significant results of the ALE meta-analysis of emotional processing tasks (EMO). All results are displayed on the left and right lateral surface view, the anterior/posterior and dorsal/ventral view of the MNI single subject template. ACC: anterior cingulate cortex, AMY: amygdala, DS: dorsal striatum, FG: fusiform gyrus, INS: insula cortex, PREC: precuneus, PCC: posterior cingulate cortex, VS: ventral striatum.

Consistent activation across the emotional processing tasks was found in the amygdala (AMY) bilaterally, the ventral (VS) and dorsal striatum (DS) bilaterally, middle and superior temporal gyrus and insular cortex, anterior cingulate cortex (ACC), posterior cingulate cortex (PCC) and the precuneus, area V5 and fusiform gyrus (FG) (Figure 2, Table 2).

**Table 2:** Activation peaks of ALE meta-analysis of the neural correlates of emotional processing tasks (EMO).

Macroanatomical location	MNI coordinates			Cluster size	Maximum z-score
	x	y	z		
Left Amygdala	−22	−6	−14	11445	8.70
Left Superior Medial Gyrus	−2	50	20	3636	8.37
Right Middle Temporal Gyrus	48	−66	0	1775	8.01
Left Inferior Temporal Gyrus	−42	−62	−10	1357	7.10
Left Precuneus	−6	−54	20	495	6.12
Left Middle Temporal Gyrus	−56	−34	−4	299	4.88
Right Middle Temporal Gyrus	52	−40	4	444	5.52

All peaks were assigned to the most probable brain area by using the SPM Anatomy Toolbox.

#### ‘Default mode network’ (DMN)

**Figure 3:**
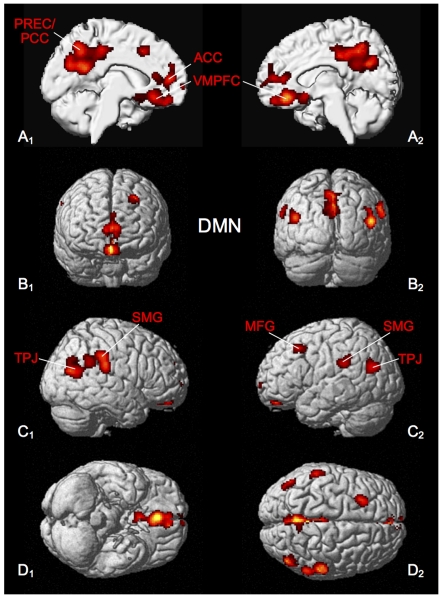
Significant results of the ALE meta-analysis of unconstrained cognition (DMN). All results are displayed on the left and right lateral surface view, the anterior/posterior and dorsal/ventral view of the MNI single subject template. ACC: anterior cingulate cortex, MFG: middle frontal gyrus, PCC: posterior cingulate cortex, PREC: precuneus, SMG: supramarginal gyrus, TPJ: temporo-parietal junction, VMPFC: ventro-medial prefrontal cortex.

Consistent task-induced deactivation was found in PCC extending into the precuneus, ACC, ventro- and dorso-medial prefrontal cortex, bilateral supramarginal gyrus, bilateral TPJ, left superior and right middle temporal gyrus, left middle occipital gyrus and left middle frontal gyrus (Figure 3, Table 3).

**Table 3:** Activation peaks of ALE meta-analysis of unconstrained cognition (DMN).

Macroanatomical location	MNI coordinates			Cluster size	Maximum z-score
	x	y	z		
Left Posterior Cingulate Cortex	−2	−54	24	2383	7.26
Right Mid Orbital Gyrus	4	34	−14	1023	8.26
Right Supramarginal Gyrus	56	−28	24	711	6.01
Left Superior Medial Gyrus	−4	52	10	723	5.64
Right Middle Temporal Gyrus	48	−70	18	442	6.29
Left Middle Occipital Gyrus	−42	−72	22	280	5.61
Left Middle Frontal Gyrus	−26	18	44	240	6.40
Left SupraMarginal Gyrus	−58	−34	28	190	4.94
Left Amygdala	−22	−4	−26	122	4.32

All peaks were assigned to the most probable brain area by using the SPM Anatomy Toolbox.

### Conjunction analyses

**Figure 4:**
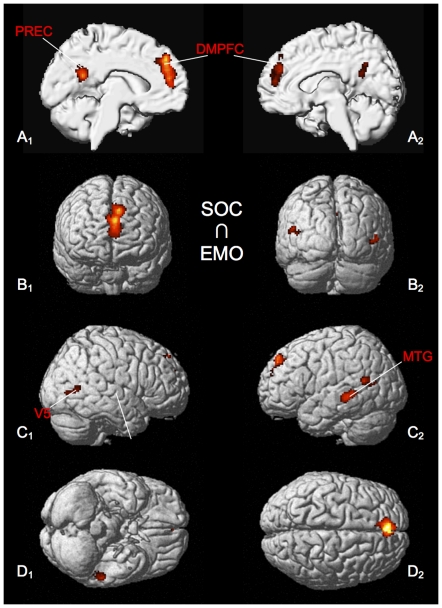
Significant results of the conjunction analysis of SOC ∩ EMO. All results are displayed on the left and right lateral surface view, the anterior/posterior and dorsal/ventral view of the MNI single subject template. DMPFC: dorso-medial prefrontal cortex, PREC: precuneus, STG: superior temporal gyrus, VS: ventral striatum.

Brain regions, which showed consistent activation across both the emotional and social-cognitive tasks (SOC ∩ EMO), comprise DMPFC, precuneus, middle and anterior superior temporal gyrus and VS (Figure 4, Table 4).

**Table 4:** Activation peaks of conjunction analysis of SOC ∩ EMO.

Macroanatomical location	MNI coordinates			Cluster size	Maximum z-score
	x	y	z		
Left Superior Medial Gyrus	−2	48	28	857	7.21
Left Precuneus	−6	−54	22	271	5.85
Left Middle Temporal Gyrus	−56	−34	3	211	4.88
Left Middle Temporal Gyrus	−50	−56	16	92	4.03
Right Middle Temporal Gyrus	52	−62	6	69	3.73

All peaks were assigned to the most probable brain area by using the SPM Anatomy Toolbox.

**Figure 5:**
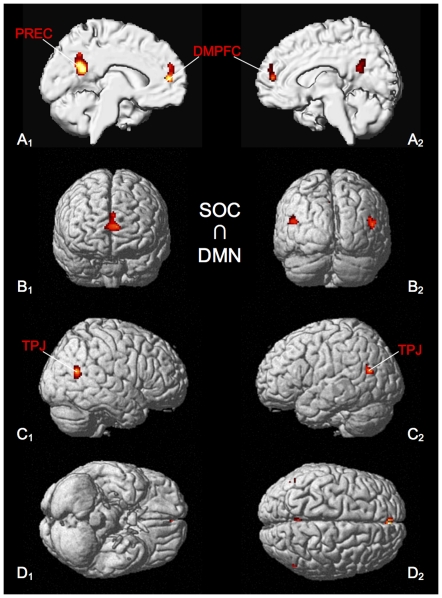
Significant results of the conjunction analysis of SOC ∩ DMN. All results are displayed on the left and right lateral surface view, the anterior/posterior and dorsal/ventral view of the MNI single subject template. DMPFC: dorso-medial prefrontal cortex, PREC: precuneus, TPJ: temporo-parietal junction.

Brain regions, which showed consistent involvement both in the social- cognitive tasks and those studies that investigated the DMN (SOC ∩ DMN), comprise DMPFC, precuneus and the TPJ bilaterally (Figure 5, Table 5).

**Table 5:** Activation peaks of conjunction analysis of SOC ∩ DMN.

Macroanatomical location	MNI coordinates			Cluster size	Maximum z-score
	x	y	z		
Left Precuneus	−6	−54	24	401	5.91
Left Superior Medial Gyrus	−2	52	14	195	4.84
Right Middle Temporal Gyrus	52	−62	16	101	3.98
Left Middle Temporal Gyrus	−46	−66	18	69	4.15

All peaks were assigned to the most probable brain area by using the SPM Anatomy Toolbox.

**Figure 6:**
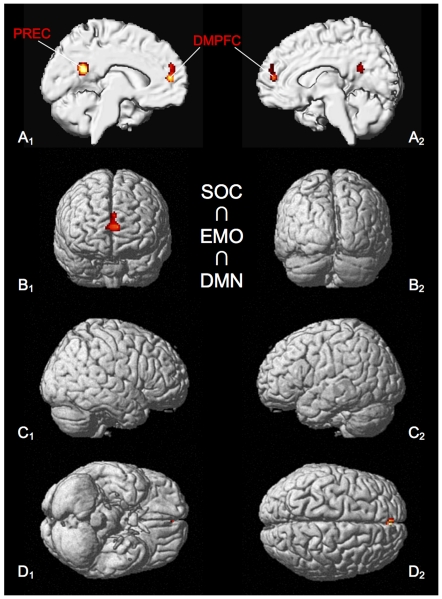
Significant results of the conjunction analysis of SOC ∩ EMO ∩ DMN. All results are displayed on the left and right lateral surface view, the anterior/posterior and dorsal/ventral view of the MNI single subject template. DMPFC: dorso-medial prefrontal cortex, PREC: precuneus.

Using a triple conjunction analysis, we formally tested for brain regions, which show consistent involvement across all different types of studies, i.e. social-cognitive, emotional processing and resting state experiments (SOC ∩ EMO ∩ DMN). Results of this analysis demonstrate the involvement of DMPFC and the precuneus across these three domains (Figure 6, Table 6).

**Table 6:** Activation peaks of conjunction analysis of SOC ∩ EMO ∩ DMN.

Macroanatomical location	MNI coordinates			Cluster size	Maximum z-score
	x	y	z		
Left Precuneus	−6	−54	22	251	5.85
Left Superior Medial Gyrus	−2	52	14	179	4.84

All peaks were assigned to the most probable brain area by using the SPM Anatomy Toolbox.

To assess whether these are the same regions as the ones reported to underlie introspective abilities (Fleming et al. 2010), we calculated a minimum conjunction analysis across the thresholded maps from the triple conjunction analysis and results from the morphometric study by Fleming and colleagues (2010). This additional analysis, indeed, demonstrates involvement of DMPFC (MNI coordinates: −3, 54, 21; T = 3.18) and precuneus (MNI coordinates: 2, −51, 19; T = 3.28), thereby confirming anatomical overlap between their results and the triple-conjunction of meta-analyses.

## Discussion

Using ALE-based meta-analyses of neuroimaging studies from the BrainMap database, we were able to demonstrate consistent patterns of activations and deactivations across different types of experimental paradigms. Providing such a quantitative assessment of convergence across studies crucially goes beyond what qualitative reviews of studies investigating social cognition and emotional processing can provide. Furthermore, our approach allows to formally investigate commonalities in the neural networks subserving different types of tasks by applying conjunction analyses, which test for convergence across different pairs and across all three individual meta-analyses. By using the latter type of analysis, we can demonstrate overlap of task-related signal change in dorso-medial prefrontal and medial parietal cortex, brain regions that have recently been linked to introspective abilities.

### ‘Social-cognitive network’ (SOC)

The meta-analysis targeting consistent activations across studies exploring the neural correlates of social cognition revealed activations in the DMPFC, the precuneus, the TPJ and anterior temporal cortex (Figure 1), which is in very good agreement with previous descriptions of the neural network subserving social cognition (e.g. [3], [27], [35]).

More specifically, DMPFC has commonly been related to mental state attribution or ‘mentalizing’ as e.g. classical false-belief tasks and stories, which require (cognitively) taking someone else's perspective, have been shown to result in a differential increase of neural activity in this area [36]. Activity of ATC has been related to the understanding of social concepts [37], [38] while activation of more posterior temporal areas and the temporo-parietal junction (TPJ), in particular, have been connected to the perception of biological motion cues, but also the understanding of intentions and perspective-taking behavior [27], [39], [40]. Weighting these putative functional contributions, activations of the TPJ have been understood as contributing to the inference of temporary goals and intentions at a perceptual level, while DMPFC has been discussed in terms of an integration of social information across time and at a more abstract level [26]. Recruitment of the precuneus (and PCC) have been related to the generation of “internally directed thoughts” [41], [42], which is likely to be important for our understanding of the – at least in part – unobservable aspects of another person's mental life. Similarly, the role of medial parietal lobe in autobiographical memory has been emphasized by Spreng and Mar, who see overlapping activations in mentalizing and memory tasks as an indication for the importance of the role of memory for social cognition, during which the integration of both personally and interpersonally available information may be crucial [2].

### ‘Emotional network’ (EMO)

Our analysis of consistent brain activations for emotional processing revealed a highly significant, bilateral pattern of activation, which comprises the AMY, the VS and DS, the ACC/DMPFC and PCC/precuneus, area V5 and insular cortex (Figure 2).

Co-activations of ACC and anterior insular cortex bilaterally have commonly been described as a “salience network” believed to be involved in the attribution of salience to both internal events and extrapersonal stimuli, thereby guiding behavior [43]–[45]. Also, this network has been implicated in the experience of and empathy for pain [46], [47]. More specifically, anterior insula cortex may be involved in re-mapping of interoceptive afferences, thus contributing to integrative representations of feelings from the body [48], [49]. ACC has classically been described as a brain region involved in the processing of cognitive control and conflict monitoring [50], [51], but more recent data suggests a role as a “hub where information about reinforcers can be linked to motor centers responsible for expressing affect and executing goal-directed behavior” [52].

Additionally, our analysis revealed involvement of the bilateral amygdala, a brain region whose primary function appears to reside in signaling what is important and to then modulate appropriate (response) processes to deal with the challenges and opportunities present in a given situation [53], [54]. Neuroanatomically, this appears to be reflected by a multitude of interconnections of the amygdala and other brain areas [55]–[57]. Consistent with the suggestion of the amygdala as an emotional significance detector, studies have indicated that intact functioning of the amygdala is important for the generation of adaptive responses [58] and for the attending of emotionally relevant information [59], [60]. In line with these views, the anatomical volume of the amygdala has been shown to correlate with the size and complexity of social networks in adult humans [61].

Finally, our meta-analysis also provides evidence for the recruitment of the ventral and dorsal striatum during emotional tasks: while the ventral striatum has been described as a core node of the neural network of reward, especially involved in the anticipation and experience of the hedonic aspects of rewards [62], [63]. The dorsal striatum, on the other hand, has been linked to reward-related aspects of action selection and action release mechanisms, thereby contributing to reward-based decision-making (e.g. [64]).

### ‘Default mode network’ (DMN)

The meta-analysis that explored consistent, task-induced deactivations across a large number of neuroimaging studies from different task domains demonstrated involvement of ACC/DMPFC and PCC/precuneus, VMPFC and TPJ bilaterally (Figure 3). These results are in line with findings from our own previous investigation of task-induced deactivations, but also other analyses, which have repeatedly demonstrated that these brain regions exhibit task-related decreases in neural activity during tasks which require engagement with external stimuli [5], [12], [65], [66].

### Convergence across the different pairs of meta-analyses

The conjunction analysis of social-cognitive and emotional processing (SOC ∩ EMO) demonstrates involvement of DMPFC, the precuneus, middle and anterior superior temporal gyrus and the ventral striatum (Figure 4). Consistent with intuition of commonalities of the phenomenal content and construct similarities between social-cognitive and emotional processes, our results, thus, suggest a common neural network across these two types of experiments. As many regions in this network (DMPFC, PCC/precuneus, TPJ) have been implicated as a core network for social cognition, we would interpret the observed convergence with emotional processing as strong evidence for the fundamental contribution of affective information to social cognition [3], [26]. Furthermore, our analysis reveals consistent overlap in the ventral striatum (Figure 4 A_1_), which has been described as a core element of the functional neuroanatomy of reward and is thought to be involved in the anticipation and experience of the hedonic aspects of rewards (e.g. [62]). The current data is thus in line with recent evidence suggesting that being able to successfully initiate and lead social interactions recruits this subcortical brain region and leads to positive subjective experiences [63]. Also, higher activity in this brain region in response to social stimuli (such as faces) has been shown to correlate with increases in reaction time needed by participants to disengage from a social stimulus [51].

Results of the conjunction analysis between social cognition and the task-related deactivations (SOC ∩ DMN), demonstrate involvement of DMPFC, precuneus and the TPJ bilaterally (Figure 5). Based on this striking convergence between the resting state and the social cognition network, one could be tempted to speculate about similarities in the associated cognitive processes. Indeed, different strands of evidence exist to suggest that human beings may have a predisposition for thinking about themselves and others, to which they return when they have nothing else to think about (see [5]). Recent findings reported by Andrews-Hanna and colleagues, indeed, lend empirical support to this idea: In their study, the authors manipulated factors that promote internally directed, spontaneous cognition separately from those that influence attention to external task demands. Results demonstrate that areas of the DMN increased their activity when spontaneous cognition was maximized [67]. Future research could extend the use of thought probes and experience sampling during resting state fMRI measurements in combination with multivariate pattern analysis techniques to investigate such matters in further detail [68], [69]. The latter approach may also be useful to assess the commonalities of the cognitive correlates and region-specific activity changes as well as possible similarities concerning the neurocomputational formats of social and unconstrained cognition (cf. [70]).

### Convergence across all three meta-analyses

As the key finding of our study, the conjunction analysis performed by including the results of all individual ALE meta-analyses (SOC ∩ EMO ∩ DMN) provides compelling empirical evidence for a shared neural network, which comprises the precuneus and DMPFC (Figure 6). This analysis was performed based on the idea that in cognitive terms a commonality may exist between all three types of states, namely that they may all be characterized by introspective processing, which could be -at times- equally relevant for coming to terms with one's own or other people's states. Consistently, the results of the triple conjunction analysis (Figure 6) converge with findings of a recent study, which has related structural brain differences in DMPFC and the precuneus to introspective abilities ([16]; see Figure 3a). Here, the dorsal part of the MPFC seems to be specifically related to prospective metacognition [71] – indeed, thinking of oneself in the context of one's future behavior would be crucial to our capacity at social interaction. Consistent with the suggestion of these two brain regions being relevant for introspection, activity changes in precuneus have been observed when individuals direct their attention internally [72]. Similarly, the DMPFC has been related to the ability of generating stimulus-independent thoughts to explain aspects of another person's mental states that might not be directly observable in their behavior [73]. Both regions have, therefore, been implicated in the generation of stimulus-independent thoughts [74], [75]. Furthermore, recent work indicates that this midline network could be related to automatically tagging the ‘self-relevance’ of stimuli (e.g. [67], [76], [77]). If this was the case, the network might play an interestingly dual role in being part of and shaping bottom-up processes, while at other times also contributing to the top-down regulation of social behavior by means of introspective processes. In other words, it may serve as an interface between neural networks, which subserve internally as compared to externally directed cognition [42], [78]. Thereby, the idea of introspective processes being related to activity in anterior medial prefrontal cortex may help to reconcile previous controversy [74], [79], as introspection can be applied to both external and internal stimuli.

Findings, which suggest that introspection can be enhanced via social interaction [80], also prompt the intriguing question of the interrelation of socio-emotional and introspective abilities and their respective neural correlates. Evidence from developmental psychology has been taken to suggest that introspective competence emerges through the infant's social interactions with others by relating one's own states with those of others [11]. However, a more radical approach holds that it may be only via social interaction and in virtue of the fact that we are constantly trying to model other minds in interaction that we learn to be conscious and develop both an understanding of ourselves [81] (Carruthers 2009) and a conscious percept of the world at all [82], [83]. In line with the suggestion that activity changes in the cortical midline structures may primarily be forged during social interactions before they may be brought under top-down control and used for ‘offline’ social cognition, a recent fMRI study was able to demonstrate the recruitment of DMPFC and PCC during (gaze-based) social interactions including joint attention in the absence of an explicit mentalizing task [63]. Consequently, one could speculate that it might be exactly via such (gaze-based) social interactions, which include deictic elements, that children's introspective competence emerges. On the neural level, we suggest that activity changes in medial prefrontal cortex-posterior cingulate connectivity - initially not found in infants [84] - are a likely candidate in terms of the neural correlate of such changes. The study by Bahrami and colleagues [80] further indicates that interaction-based changes of introspective abilities continue to be effective during adulthood.

Taken together, or results suggest that on the neural level, introspective, social and affective processes converge and rely upon recruitment of a network that comprises dorso-medial prefrontal and medial parietal cortex.

### Conclusions & Outlook

In this study, we took a meta-analytic approach to delineating brain regions, which consistently show *activations* during social cognition and emotional processing and *deactivations* across a wide range of experimental tasks. By applying conjunction analyses, we demonstrated a close convergence of the brain regions involved. These results provide robust empirical evidence for a shared neural network that underlies emotional processing, social and unconstrained cognition, which localizes to the precuneus and anterior medial prefrontal cortex. These two regions are known to be critical hubs in the neurofunctional architecture of the human brain [1], [41], [85]–[89]. Moreover, these regions have been shown as closely related to introspective abilities [16]. Crucially, comparing the results of our triple conjunction analysis to the findings by Fleming and colleagues demonstrates significant anatomical overlap. Consequently, we argue that these findings are consistent with the idea that a ‘common denominator’ may exist in cognitive terms, which could consist in introspective processes. By making use of these, we may become aware of our own or others' states, equally relevant for social cognition, emotional processing and unconstrained thought [5], [24].

Future research into the functional and effective connectivity of the different nodes of this shared neural network and its susceptibility to intervention and plasticity during social interactions are likely to be informative concerning the exact functional roles of the different brain regions. One important question our findings raise is whether social cognition, emotional processing, and resting state cognition simply recruit brain structures that subtend conscious introspection or meta-cognitive abilities, or whether they are in fact instrumental in the shaping of such introspective abilities. One possibility is that in social or emotional contexts, not only adequate behavior is important, but that the successful prediction and monitoring of the outcomes of this behavior is equally essential. Such re-description of one's behavior could turn out to be exactly the mechanism that underlies introspection [90]–[92].
